# How uncommon is the occurrence of orthokeratinized odontogenic cyst in the maxilla? A systematic review and a new case report

**DOI:** 10.4317/jced.63735

**Published:** 2026-02-26

**Authors:** Dhara Schrok Monteiro de Ataide, Kaique Alberto Preto, Gabriela Lopes dos Santos, Beethoven Estevão Costa, Roberto Yoshio Kawakami, Denise Tostes Oliveira

**Affiliations:** 1 Department of Surgery, Stomatology, Pathology, and Radiology, Area of Pathology, Bauru School of Dentistry - University of São Paulo, Bauru, São Paulo, Brazil; 2 Department of Dentistry, UNISAGRADO, Bauru, São Paulo, Brazil; 3 No affiliation

## Abstract

**Background:**

The orthokeratinized odontogenic cyst (OOC) is a benign intraosseous lesion, typically found in the posterior mandible in male patients in the third and fourth decades of life. Although there has been a growing number of documented cases in the last decade, the prevalence and unique characteristics of maxillary OOC remain poorly understood.

**Material and Methods:**

To assess how uncommon is OOC occurrence in the maxilla, a systematic review of the literature was conducted using the major electronic databases and rigorous criteria, including a new case report involving a young male patient.

**Results:**

A total of 36 OOC located specifically in the maxilla was analyzed. The results demonstrated that OOC in the maxilla is a rare finding. Interestingly, the majority of instances are associated with an impacted tooth, mainly unerupted third molars and canines, a pattern previously unexplored in the literature. An isolated occurrence of an OOC in the posterior maxilla without involvement of a third molar tooth, as observed in this present case, is relatively uncommon and has been reported in only five other cases. Careful enucleation and curettage with removal of the impacted teeth, when present, followed by histopathological analysis, has proven to be the most effective approach for accurate diagnosis and clinical success.

**Conclusions:**

This study reinforces that orthokeratinized odontogenic cysts should be included in the differential diagnosis of intraosseous lesions in the maxilla, especially when associated with unerupted third molars and canines, which may clinically resemble dentigerous cysts.

## Introduction

The orthokeratinized odontogenic cyst (OOC) was first proposed as clinicopathologic variant of the odontogenic keratocyst (OKC) in the mid-1980s by Wright JM, due to its less aggressive biological behavior, lower recurrence rate, and lack of association with nevoid basal cell carcinoma syndrome (1). Despite Wright´s early proposal, the lesion was officially classified as a distinct pathological entity in the fourth edition of the World Health Organization Classification of Head and Neck Tumours, published in 2017 (2). Since then, the OOC has been recognized as a rare, benign intraosseous lesion characterized by a cystic cavity lined by orthokeratinized stratified squamous epithelium (2 , 3). The etiopathogenesis of the OOC is believed to originate from the remnants of the dental lamina (4), which may explain its frequent association with impacted or unerupted teeth (3 , 5). The lesion typically occurs in the posterior mandible and predominantly affects male patients in the third to fourth decades of life (3 , 5). Clinically, the OOC is generally asymptomatic and exhibits an indolent behavior, often discovered incidentally on radiographic examination or presenting as a localized, well-defined swelling (3 , 5). To investigate how uncommon the occurrence of OOC in the maxilla is, the present study was conducted, including a new case report involving a young male patient, accompanied by a systematic review of the literature. The clinical, radiographic and histopathological characteristics of the OOC are discussed, with particular emphasis on its presentation in the maxilla.

## Material and Methods

**Figure 1:**
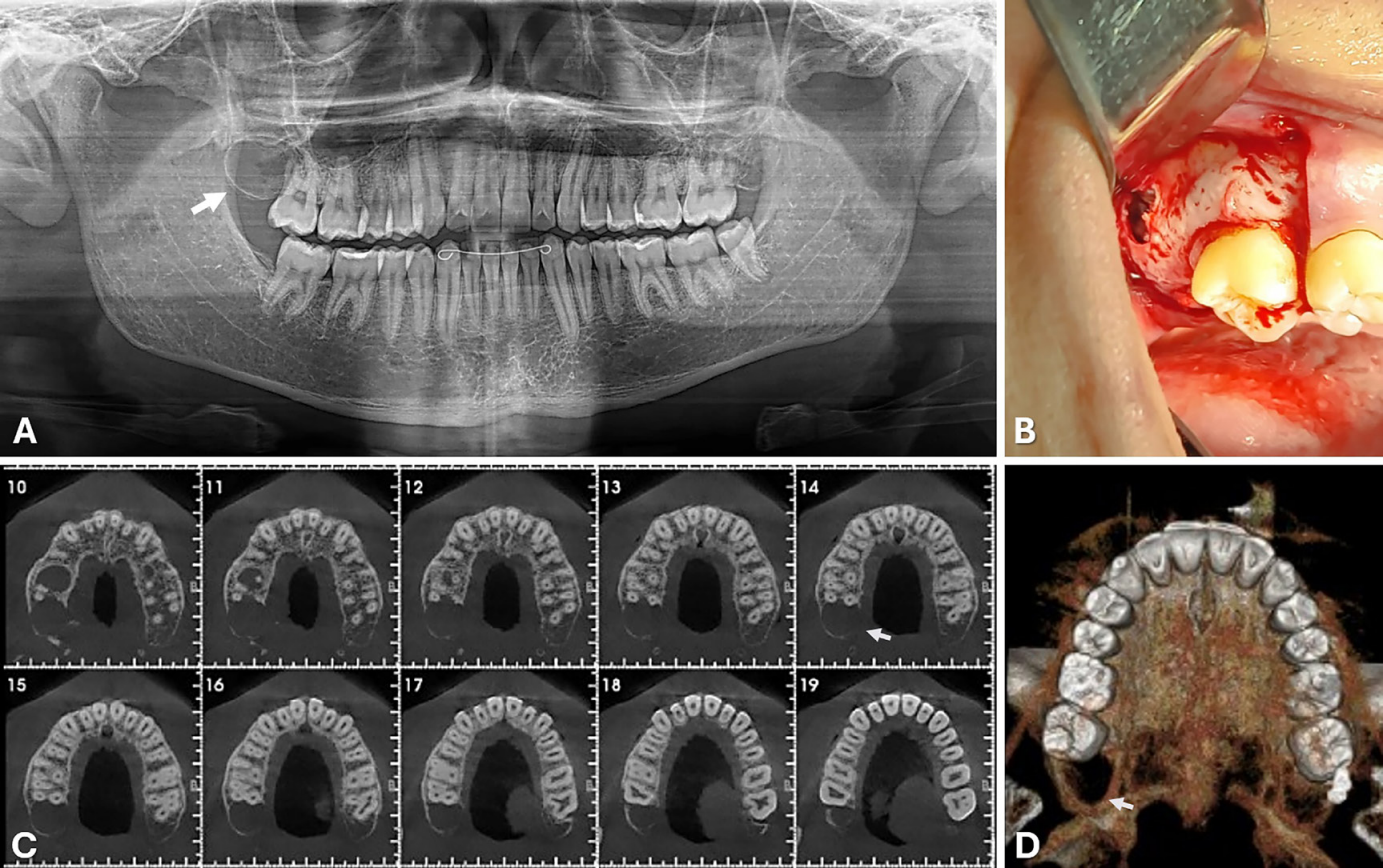
A: Clinical and imaginological features of the orthokeratinized odontogenic cyst in the posterior maxilla. Panoramic radiography revealed an unilocular, circular radiolucency surrounded by a radiopaque halo in the region of the left maxillary second molar (white arrow). B: Surgical access view demonstrating the precise localization of the cyst and a vestibular cortical rupture. C: Cone-beam computed tomography (CBCT), in axial reconstructions, confirmed the lesion’s proximity to the roots of the second molar, with no signs of root resorption and palatal and vestibular cortical thinning (white arrow). D: A three-dimensional reconstruction of the maxilla clearly delineated the well-defined lesion (D).

A 17-year-old Caucasian male was referred to the dentist for the evaluation of a radiographic finding: an intraosseous, asymptomatic lesion located in the right maxillary tuberosity, with an undetermined duration of development. Clinical examination revealed no alterations in the affected region. His medical history was unremarkable and there was no history of previous surgical intervention involving the area of the maxillary right third molar, which was associated with the lesion. Panoramic radiography revealed a well-defined, unilocular, circular radiolucency surrounded by a radiopaque halo (Fig. 1A).

1

**Figure 2:**
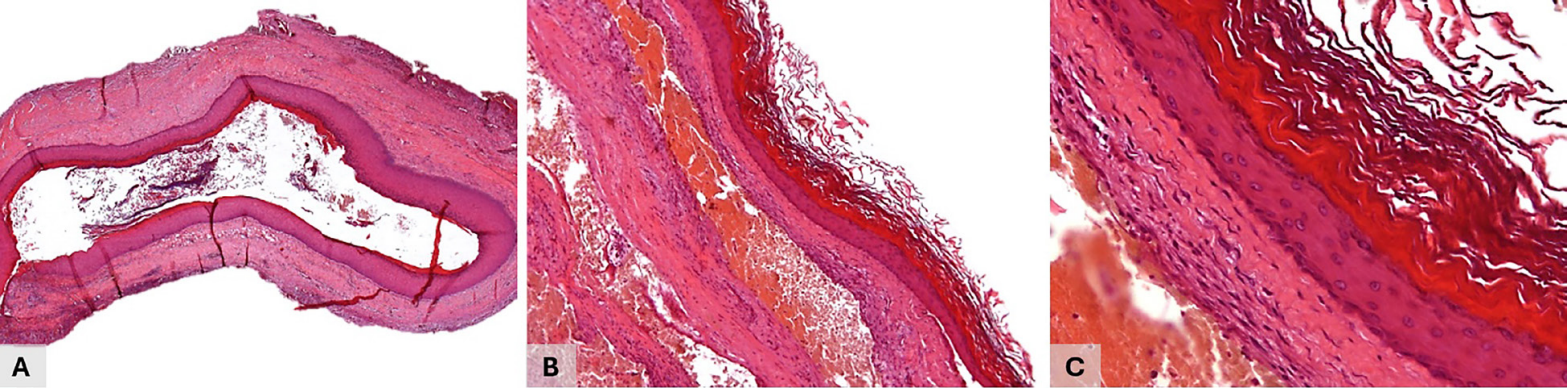
A: Microscopic features of the orthokeratinized odontogenic cyst in the posterior maxilla. The lesion exhibited a thin, highly vascularized fibrous capsule adjacent to a virtual cystic cavity, B: Epithelium lined by orthokeratinized stratified squamous epithelium lacking epithelial ridges, and characterized by a prominent granular cell layer and a basal cell layer with hyperchromatic nuclei. C: The cystic lumen contained numerous concentric lamellae of keratin. (H&amp;E stain. Original magnifications: A, 25×; B, 100×; C, 400X).

Notably, the tooth germ of the maxillary third molar was absent on imaging. Cone-beam computed tomography (CBCT) confirmed the lesion's proximity to the roots of the second molar, without any signs of root resorption (Figs. 1C,D). The presumptive diagnosis was an odontogenic cyst or tumor. Surgical enucleation of the lesion was performed under local anesthesia, administered via terminal infiltration and posterior superior alveolar nerve block techniques. Surgical access was achieved through a relieving incision on the mesial, intrasulcular, and distal aspects of the second molar, followed by elevation of a mucoperiosteal flap (Fig. 1B). The procedure was uneventful, and the surgical specimen was submitted for histopathological analysis. Microscopically, the lesion exhibited a virtual cystic cavity lined by orthokeratinized stratified squamous epithelium lacking epithelial ridges and characterized by a prominent granular cell layer and a basal cell layer with hyperchromatic nuclei (Fig. 2).

2

**Figure 3:**
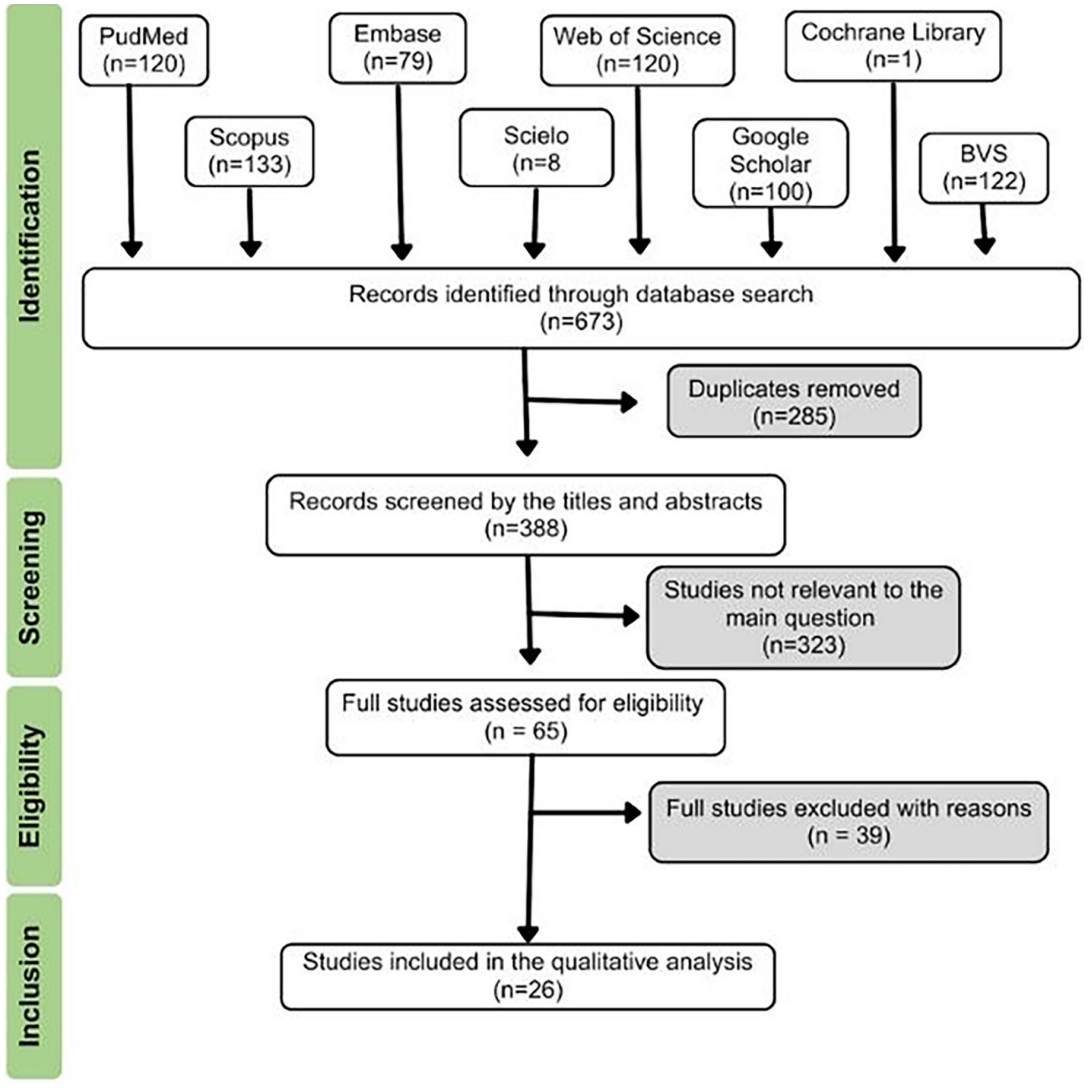
Flowchart screening the steps for the articles included in this systematic review.

Beneath the epithelial lining, a thin, highly vascularized fibrous capsule was noted. The cystic lumen contained numerous concentric lamellae of keratin. The diagnosis of orthokeratinized odontogenic cyst was established. After two-years of follow-up, the patient exhibited normal healing with no clinical and radiographic evidence of recurrence. Systematic review of the literature Study design This systematic review was conducted in accordance with PRISMA 2020 statement guidelines (Preferred Reporting Items for Systematic Reviews and Meta-Analysis) (6) as described in Fig. 3.

3

The protocol was registered in PROSPERO (CRD420251156287). The study aimed to address the following question: How uncommon is the occurrence of the orthokeratinized odontogenic cyst in the maxilla? The PICOT framework was used to structure the study. Search strategies The literature search was performed in the following electronic databases: PubMed/MEDLINE, Scopus, Web of Science, Cochrane Library, SciELO, and EMBASE. Additionally, the gray literature was manually searched using the Virtual Health Library (BVS) and Google Scholar (the first 100 results). The search strategy was adapted for each database. All searches were conducted on September 18, 2025. There were no limitations on language or publication year. The following search strategy was carried out using the keywords: "orthokeratinized odontogenic cyst" or "orthokeratinized odontogenic keratocyst" or "orthokeratinized keratocystic odontogenic tumour" and "maxilla". Eligibility Criteria The inclusion criteria were based on the Patient, Intervention, Comparison, Outcome, and Study design (PICOT) strategy: Population: Patients with orthokeratinized odontogenic cyst located exclusively in the maxilla. Intervention: Conclusive histopathological diagnosis after biopsy. Comparison: None. Outcome: Prevalence and characterization (clinical, radiographic, and histopathological features). Types of study: Cross-sectional studies, case series, case reports, letters to the editor, and short communications. The exclusion criteria were: Studies reporting on anatomical sites other than the maxilla or without histopathological confirmation. Pre-clinical and in vitro studies. Clinical studies or case series lacking key individualized information about patients' clinicopathological features (including sex, age, lesion location and histopathological informations), treatment, or clinical outcome. Study Selection All retrieved studies eligible for inclusion were imported into the reference management tool EndNote Web database and duplicates were removed. In the first phase of study selection, two independent authors screened the titles and abstracts of the studies for relevance. Any disagreements were resolved through discussion, and an expert in Oral Pathology was consulted if necessary. In the second phase, the same authors independently read the full texts of the studies selected in phase 1, applying the inclusion and exclusion criteria. Disagreements were resolved through discussion among the authors and, when necessary, by consulting a third reviewer. In addition to the literature review, the present case report was also included in the study. Data Extraction Two authors extracted relevant information from the studies into a spreadsheet. The collected data included: study design, country, number of patients, demographic characteristics (sex and age), and clinicopathological features (clinical description, anatomical location, histopathological description and imaging exams). A total of 26 studies, including 36 OOCs located in the maxilla, were selected for qualitative analysis. The findings are summarized in Table 1 (4 , 7 - 31).

Table 1

Statistical analysis Data analysis was conducted using IBM SPSS Statistics, version 20 (Chicago, USA). The analysis included descriptive statistics based on mean, standard deviation and percentage values.

## Results and Discussion

Although OOC is well recognized as a rare benign lesion, accounting for about 1% of all odontogenic cysts, with the mandible being affected more than twice as often as the maxilla (2 , 3), its clinical, radiographic and post-treatment characteristics, particularly in the maxilla, remain poorly explored in the literature. Since the systematic review of orthokeratinized odontogenic cysts (OOCs) conducted by MacDonald-Jankowski et al. in 2010 (32), the publication of new case reports and clinicopathological studies has significantly advanced knowledge of this uncommon cyst (7 - 28). In the present study, a systematic literature review identified well-documented OOC cases published to date, with 36 cases affecting the maxilla, a finding that highlights not only the rarity of this anatomical location, but also the relevance of further examining such cases to deepen insights into their clinical presentation, imaging findings, and histopathological features. This review results confirmed that most cases of maxillary OOCs occurred in males (70.59%), resulting in a male-to-female ratio of 2.4:1 (Table 1). The patients' ages ranged from 18 to 65 years, with a predominance in the third (21 to 30 years - 47.06%) followed by the fourth (31 to 40 years - 23.53%) decades of life, and a mean age of 33.35 years. Interestingly, the present case involved a 17-year-old male, and only two other published cases have occurred during the second decade of life (13 , 19). Furthermore, the posterior maxillary region, including the present case, was the most affected site, representing 50% of reported maxillary OOCs, with two cases presenting bilateral posterior involvement (19 , 24). On the other hand, the anterior (25%) and anteroposterior (25%) regions of the maxilla exhibited a significant prevalence for this odontogenic cyst, as summarized in the Table 1. According to the imaging findings, maxillary OOCs typically appear as unilocular, well-circumscribed radiolucent or hypodense lesions (97.2%). In terms of dental involvement, 62.8% of OCCs were associated with an impacted tooth. Among these, 54.5% involved unerupted third molars and, notably, 27.2% were associated with unerupted canines, underscoring the relevance of canines in the presentation of maxillary OCCs (Table 1). An isolated occurrence of an OOC in the posterior maxilla without involvement of a third molar tooth, as observed in this present case, is relatively uncommon and has been reported in five other cases in the literature (4 , 11 , 14 , 20 , 29). Due to their overlapping clinical and imaging features with other odontogenic maxillary lesions, OOCs are frequently misdiagnosed as dentigerous cysts (44.4%), odontogenic keratocyst (19.4%), adenomatoid odontogenic tumor (8.3%), unicystic ameloblastomas (2.7%) or inflammatory periapical lesions (25%), depending on the lesion's location and extent (Table 1). Nonetheless, several studies have reported a considerable number of symptomatic cases, particularly when secondary infection is present. In such instances, clinical signs such as swelling and, occasionally, pain may occur (5 , 32). These findings highlight the need for clinical vigilance regarding potential inflammatory signs, even in lesions traditionally considered asymptomatic, as observed in the present case. A total of 47.2% OOC lacked long-term follow-up data, limiting a reliable assessment of recurrence rates. Nevertheless, 44.4% of cases showed no recurrence, with a mean time to relapse of approximately 31.5 months. Only one case of the maxillary OOC in a female patient in the fourth decade of life had a recurrence (18), as described in the Table 1. As a result of their indolent behavior and low recurrence rates, aggressive treatment modalities such as resection, enucleation followed by peripheral ostectomy, or chemical cauterization with Carnoy's solution, previously used in the management of keratocystic odontogenic tumors, are generally unnecessary in most cases of OOC (5 , 8 , 32). As demonstrated in the present case, careful enucleation and curettage with extraction of the impacted teeth, when present, have proven to be an effective approach, sufficient to significantly reduce the already low recurrence rates (5 , 9). Orthokeratinized odontogenic cysts typically present as isolated lesions; however, the literature reports 16 cases of multiple lesions (8 , 33 - 35), some of those were involving maxilla (33) and none of these cases were associated with Gorlin syndrome. Although no syndromic association was observed in the reviewed multiple OOCs, loss of heterozygosity involving the PTCH gene has been reported in sporadic cases, suggesting a possible shared molecular pathway with the syndromic variant (24 , 36). Microscopically, OOCs are characterized by a thin and uniform epithelial lining, typically composed of 4 to 8 layers of stratified orthokeratinized squamous epithelium (3 - 5), as observed in the present case (Figs. 2A and 2B). This lining displays a well-developed granular cell layer and a basal layer consisting of discrete or scarcely perceptible cuboidal and/or flattened squamous cells (3 , 4 , 10). Unlike odontogenic keratocyst, OOCs usually do not exhibit prominent palisading or hyperchromatism of basal cell nuclei (Fig. 2C). The underlying connective tissue capsule is predominantly fibrous in nature and may occasionally exhibit focal areas of chronic inflammatory infiltration. The cystic lumen typically contains abundant lamellated orthokeratin debris, as showed in Figure 2 (3 - 5 , 10 , 19). In rare instances, focal areas of parakeratinized or non-keratinized epithelium may be present, usually in association with an underlying inflammatory response (19 , 25). The occurrence of intramural microcysts or proliferative epithelial islands is uncommon, which may partially account for the low recurrence rate associated with this lesion (3 - 5 , 19 , 32).

## Conclusions

In conclusion, orthokeratinized odontogenic cysts (OOCs) are rare in the maxilla, as demonstrated in this systematic review of the literature. Most reported cases occurred predominantly in males during the third and fourth decades of life and were associated with impacted teeth, particularly unerupted third molars and canines. Clinically, OOCs occurring in the maxilla are often misdiagnosed as dentigerous cysts, making histopathological analysis essential for an accurate diagnosis. Management of this odontogenic cyst generally consists of curettage with removal of the associated impacted teeth, and recurrence is considered extremely rare. This study reinforces that orthokeratinized odontogenic cysts should be included in the differential diagnosis of intraosseous lesions in the maxilla, particularly when associated with unerupted teeth such as third molars and canines which may clinically resemble dentigerous cysts.

## Figures and Tables

**Table 1:** Table Published clinical data of orthokeratinized odontogenic cyst located in maxilla.

First Author, Year	Age	Sex	Location in maxilla	Impacted Tooth	Clinical Diagnosis	Recurrence (time)
Ahamed et al., 2025	34	F	Posterior	Yes (3 molar)	Dentigerous cyst	NI
Dineshkumar et al., 2024	25	M	Anterior	No	Dentigerous cyst, OKC	No (2 months to 7 years)
	30	M	Anteroposterior	No	Infected Dentigerous cyst	NI
Guruprasad, Aravind, Ramadevi, 2024	22	F	Anteroposterior	Yes (canine)	Dentigerous cyst	No (1 year)
Lee et al., 2024	24	M	Posterior	Yes (3 molar)	Dentigerous cyst	NI
Hwang et al., 2023	26	M	Posterior	No	NI	NI
Metgud et al., 2023	21	F	Posterior	Yes (3 molar)	Dentigerous cyst	NI
	65	M	Anterior	Yes (mesiodens)	Dentigerous cyst	NI
	22	F	Anterior	Yes (canine)	Dentigerous cyst, AOT	NI
Mishra, Bhatt, Singh, 2022	18	F	Posterior	Yes (3 molar)	OKC	NI
Yeh et al., 2021	50	F	Posterior	No	Residual cyst	NI
Sameerudeen et al., 2021	25	M	Anteroposterior	Yes (canine)	Dentigerous cyst, AOTOKC, Unicystic ameloblastoma	No (1 year)
Behrad et al., 2021	50	M	Anterior	No	NI	NI
Joseph, Dungarwalla, Jones, 2021	29	F	Posterior	Yes (3 molar)	Dentigerous cyst	No (11 months)
Mahdavi et al., 2021	31	F	Anterior	No	NI	R (10 months)
	50	M	Posterior	Yes (3 molar)	NI	NI
Crane et al., 2020	23	M	Posterior	Yes (3 molar)	NI	No (4 years)
	20	M	Bilateral Posterior	Yes (bilateral 3 molar)	Dentigerous cyst, OKC	No (2 years)
Chien et al., 2019	39	M	Posterior	No	Residual Dentigerous cyst	NI
Naus et al., 2019	30	M	Posterior	Yes (3 molar)	NI	No (NI)
Kamarthi et al., 2016	64	M	Anteroposterior	No	Residual cyst + proliferative verrucous leukoplakia	No (6 months)
Shetty et al., 2016	50	M	Anteroposterior	Yes (canine)	NI	NI
Cheng et al., 2015	23	M	Bilateral posterior	Yes (bilateral 3 molar)	Multiple OKCs	No (14 months)
Servato et al., 2014	40	F	Anterior	No	Radicular cyst	No (2 years)
Bhasin, Pathak, Puttalingaiah, 2014	36	M	Anterior	Yes (canine)	Dentigerous cyst, AOT	No (4 weeks)
Kulkarni et al., 2013	26	M	Anteroposterior	Yes (canine)	OKC	NI
Kotwaney and Shetty, 2013	32	M	Anterior	Yes (central incisor)	Radicular cyst, Traumatic bone cyst	NI
Li et al., 1998	27	F	Posterior	No	Radicular cyst	No (9 year)
Vuhuahula et al., 1993	21	M	Anteroposterior	Yes (NI)	Dentigerous cyst	No (4 years)
	41	M	Anteroposterior	Yes (supernumerary)	Dentigerous cyst	No (4 years)
	21	M	Anteroposterior	No	Radicular cyst	No (4 years)
	38	M	Posterior	No	Residual cyst	No (4 years)
Siar and Ng, 1987	40	M	Posterior	NI	Infected dental cyst	NI
Hancock, Brown, Hartman, 1986	41	M	Anterior	No	NI	NI

Legends: M: male; F: female; NI: not informed; R: recurrence; OKC: odontogenic keratocyst; AOT: adenomatoid odontogenic tumor.
